# Proteome analysis of bronchoalveolar lavage fluids reveals host and fungal proteins highly expressed during invasive pulmonary aspergillosis in mice and humans

**DOI:** 10.1080/21505594.2020.1824960

**Published:** 2020-10-12

**Authors:** Silke Machata, Mario M. Müller, Roland Lehmann, Patricia Sieber, Gianni Panagiotou, Agostinho Carvalho, Cristina Cunha, Katrien Lagrou, Johan Maertens, Hortense Slevogt, Ilse D. Jacobsen

**Affiliations:** a Microbial Immunology, Leibniz Institute for Natural Product Research and Infection Biology – Hans Knoell Institute, Jena, Germany; b Septomics Research Centre, Jena University Hospital, Jena, Germany; c Systems Biology and Bioinformatics, Leibniz Institute for Natural Product Research and Infection Biology – Hans Knoell Institute, Jena, Germany; d School of the Biological Sciences, Faculty of Sciences, The University of Hong Kong, Hong Kong, China; e Department of Microbiology, Li Ka Shing Faculty of Medicine, The University of Hong Kong, Hong Kong, China; f Life and Health Sciences Research Institute (ICVS), School of Medicine, University of Minho, Braga, Portugal; g ICVS/3B’s-PT Government Associate Laboratory, Braga/Guimarães, Portugal; h Department of Microbiology, Immunology and Transplantation, KU Leuven, Leuven, Belgium; i Clinical Department of Laboratory Medicine and National Reference Center for Mycosis, University Hospitals Leuven, Leuven, Belgium; j Department of Hematology, University Hospitals Leuven, Leuven, Belgium; k Institute for Microbiology, Friedrich-Schiller-University Jena, Jena, Germany

**Keywords:** Invasive aspergillosis, fungal pulmonary infection, *Aspergillus fumigatus*, biomarker, proteome analysis

## Abstract

Invasive pulmonary aspergillosis (IPA) is a severe infection that is difficult to diagnose due to the ubiquitous presence of fungal spores, the underlying diseases of risk patients, and limitations of currently available markers. In this study, we performed a comprehensive liquid chromatography tandem mass spectrometry (LC-MS/MS)-based identification of host and fungal proteins expressed during IPA in mice and humans. The proteomic analysis of bronchoalveolar lavage samples of individual IPA and control cases allowed the description of common host factors that had significantly increased abundance in both infected animals and IPA patients compared to their controls. Although increased levels of these individual host proteins might not be sufficient to distinguish bacterial from fungal infection, a combination of these markers might be beneficial to improve diagnosis. We also identified 16 fungal proteins that were specifically detected during infection and may be valuable candidates for biomarker evaluation.

## Graphical abstract

**Figure:**
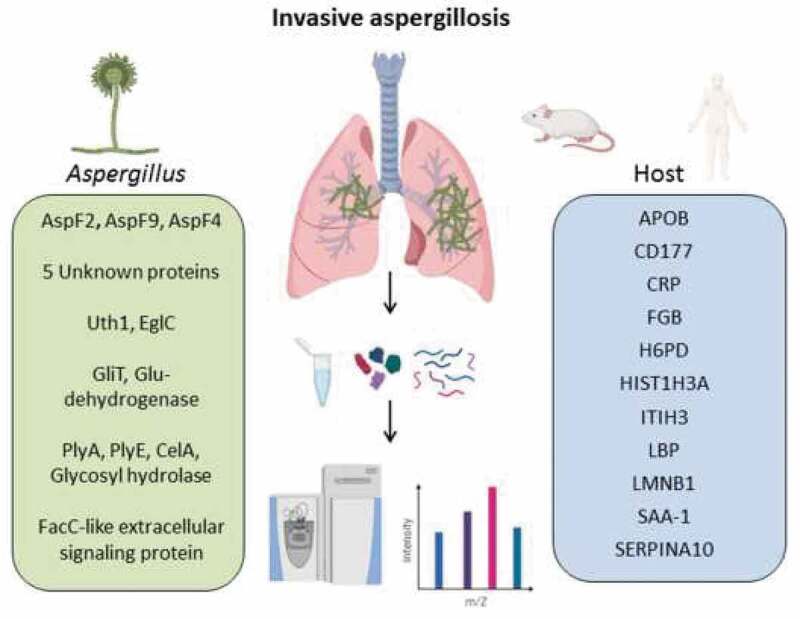

## Introduction

With over 200,000 annual cases world-wide, *Aspergillus fumigatus* is the most common cause of fungal lung infections in immunocompromised patients [1,2]. Invasive pulmonary aspergillosis (IPA) is a life-threatening complication especially in organ transplant recipients and patients undergoing hematopoietic cell transplantations with mortality rates as high as 50% despite effective treatment. Early diagnosis is considered one of the major factors improving chances of survival [1]. Challenges of diagnosis of IPA are the unspecific symptoms of fungal-mediated pneumonia and insufficient specificity or sensitivity of available tests used to screen for infection with *Aspergillus*. One particular clinical challenge relates to the differentiation of exposure or colonization with this ubiquitous fungus and infection or disease. In recent years several publications have aimed to determine secreted fungal antigens associated with *Aspergillus* invasion that could serve as potential biomarkers for faster and more sensitive and specific diagnosis of IPA [3–8]. Most of these studies used immunoblotting of fungal protein extracts derived from fungal *in vitro* cultures with patient sera containing anti- *Aspergillus* antibodies to detect infection-specific antigens [4,7]. However, the *A. fumigatus* protein profile *in-vitro* depends on the growth conditions and nutrients available in the culture medium [3] and might not adequately reflect the *in vivo* proteome [9]. Fungal protein abundance *in vivo* is, for example affected by nutrient and iron limitation, antimicrobial peptides, other host proteins such as lysozyme and collectins, and reduced oxygen availability [10,11]. Particular fungal proteins that are only produced during infection would not be identified using fungal extracts from laboratory cultures. To circumvent this problem and increase specificity for infection-relevant fungal antigens Sarfati *et al*. probed kidney extracts from intravenously infected mice with sera of IPA patients to identify infection-relevant fungal antigens [5]. However, kidney infection in the systemic mouse model differs from the conditions *Aspergillus* is facing in the respiratory tract during IPA. The leukopenic pulmonary mouse model more closely resembles the clinical symptoms and pathological changes in neutropenic human IPA patients. It furthermore allows monitoring of pulmonary fungal growth by *in vivo* imaging [12,13].

In search for IPA biomarker candidates, body fluids such as blood, urine, and bronchoalveolar lavage (BAL) fluid have been commonly used. In most studies, a combination of protein separation by 2-dimensional isoelectric focusing with mass spectrometric identification of specific protein spots was used to identify secreted fungal antigens that are unique to pulmonary infections with *A. fumigatus* [14–16]. However, this approach suffers from a major limitation: the abundance of host proteins hampers the detection of pathogen-derived proteins that are usually present at lower concentrations. Recent advances in gel-free sample preparation methods and instrumentation developments for bottom-up proteomics using liquid chromatography tandem mass spectrometry (LC-MS/MS) allow exhaustive analysis of even low abundant proteins in complex biological samples due to improvements in speed, mass resolution, sensitivity, as well as ion separation and dissociation technologies [17–20].

Here we provide a comprehensive proteomic analysis of IPA-related changes in human and murine BAL samples by using a high-resolution mass spectrometry analyzer (Orbitrap) in combination with an enhanced FASP method for increased proteome coverage [2]. We found both common and distinct alterations in the host proteome of mice and humans with IPA. Furthermore, we identified novel *A. fumigatus* -derived proteins that are abundant in infected specimens and represent candidate biomarkers for the detection of *A. fumigatus* in respiratory diseases. The data presented in this study have the potential to contribute to the development of new diagnostic tools for fungal respiratory infections.

## Materials and methods

### Ethics statements

The study, involving the collection of BAL samples and patient metadata, was approved by the Ethics Subcommittee for Life and Health Sciences (SECVS), University of Minho, Portugal (no. 126/2014), and the Ethics Committee of the University Hospitals of Leuven, Belgium. Experiments were conducted according to the principles expressed in the Declaration of Helsinki, and participants provided written informed consent. Animals were cared for in accordance with the European Convention for the Protection of Vertebrate Animals Used for Experimental and Other Scientific Purposes. All animal experiments were carried out in accordance with the German animal protection law and were approved by the responsible Federal State authority’s (Thüringer Landesamt für Lebensmittelsicherheit und Verbraucherschutz) Ethics Committee (permit no. 03-014/15).

### Human BAL fluid collection

BAL specimens were collected as previously described [21]. Briefly, specimens were collected using a flexible fiberoptic bronchoscope following local anesthesia with 2% lidocaine (Xylocaine), when infection was clinically suspected using a standard algorithm: In patients with 4–5 d of persistent fever, or with an abnormal chest X-ray, or with signs and symptoms suggestive of fungal infection (dyspnea, hemoptysis, dry cough, pleuritic chest pain, etc.) or with a positive serum biomarker, a chest CT scan was performed. Samples were obtained by instillation of a pre-warmed 0.9% sterile saline solution (20 ml twice). The sampling area was determined based on the localization of lesions on chest imaging (X-ray or computed tomography scan). All samples were stored at −80°C until use.

### Murine infection model and in vivo bioluminescence imaging

The bioluminescent *A. fumigatus* strain C3 (based on CEA10) was kindly provided by Matthias Brock (University of Nottingham, UK), stored as cryo stocks, and cultivated on maltose extract agar plates (Fluka) for 2 d at 37°C followed by 20°C for 2–3 d.

Intranasal leukopenic infection was performed as described previously [12]. In brief, spores were harvested from maltose extract agar plates using PBS containing 0.1% Tween (PBS-T) and filtered over a 40 µm cell strainer (SPL Life Sciences). Conidia were washed once in PBS-T and twice in PBS, counted using the CASY cell counter (Roche Innovatis AG) and a suspension of 2.5x 10^7^conidia/ml was prepared in PBS. Female 6–8 weeks old pathogen-free CD-1 mice (Charles River) were housed in individually ventilated cages and cared for in accordance with the European Convention for the Protection of Vertebrate Animals Used for Experimental and Other Scientific Purposes. Immune suppression was achieved by subcutaneous injection of 50 μl of 80 mg/ml hydrocortisone acetate (Sigma-Aldrich) in PBS on d 1, and peritoneal injection 140 mg/kg cyclophosphamide (Baxter) on d 3 and 1 before infection and d 2, 5, and 8 after infection if applicable. For infection, mice were anaesthetized and infected intranasally with 20 µl of PBS containing 5 × 10^5^ conidia. PBS mock-infected immune-suppressed mice were used as infection controls, infected immunocompetent mice were used as exposition controls. Fungal growth was monitored by *in vivo* imaging of bioluminescence every other day post-infection (p.i.) as described previously [12,13]. Briefly, 100 ul of 33 mg/ml D-luciferin (Synchem) in PBS was injected intraperitoneally 10 min prior imaging. Mice were anesthetized with 2.5% isoflurane (CP-Pharma) in medical oxygen using an XGI-8 anesthesia system (Caliper life science). Imaging was performed using an IVIS Spectrum (Caliper life science) and Living Image software version 3.1 (Caliper life science) by photon acquisition with small filter and binning of 4. When animals reached humane endpoints (defined by moderate dyspnea, weight loss, lethargy) or at the end of the experiment (d 10), mice were sacrificed. For collection of murine BAL fluid, a tube was inserted into the trachea and 800 µl PBS were applied while holding the mouse in an upright position. The lavage fluid was retrieved by aspiration while slowly moving the mouse into a dorsal position. 100 µl BAL fluid per sample was plated on YPD agar plates; the remainder of the samples were stored at −80°C until analysis.

### Preparation of BAL samples and LC-MS/MS

BAL samples were thawed on ice and processed without measurement or adjustment of protein content. Following thawing, samples were centrifuged at 14,000 x *g* at 4°C for 5 min before supernatants were prepared for LC-MS/MS measurement according to the eFASP method described previously [2,18]. Briefly, 50 µl BAL were mixed with 50 µl lysis buffer (100 mM Tris pH 8.5, 100 mM DTT, 2% SDS) and boiled for 3 min at 95°C. The lysate was centrifuged at 16,000 g for 5 min, the supernatant was mixed with 200 µl UA buffer (8 M Urea, 100 mM Tris pH 8.5), and applied to a Microcon YM-30-filter (Millipore, Burlington MA, USA). The sample was centrifuged at 14,000 x g for 15 min and the filter was washed with 200 µl UA buffer. 100 µl IAA solution (0.05 M iodoacetamide in UA buffer) was added followed by incubation for 20 min in the dark. Filter units were then centrifuged at 14,000 x *g* for 10 min and washed three times with 100 µl UA buffer. Following washing three times with 100 µl ABC buffer (0.05 M ammonium bicarbonate), proteins were digested with trypsin by applying 50 µl ABC/0.2% sodium deoxycholate and 1 µl 1 µg/µl trypsin (Sigma-Aldrich, Taufkirchen, Germany) to the filter unit and incubation for 16 h at 37°C. Filter units were then transferred to new collection tubes and digested peptides were retrieved by centrifugation at 14,000 x *g* for 10 min followed by a second spin with additional 50 µl of ABC. Samples were acidified by adding 0.5% formic acid and SDC was removed by three washing steps in saturated ethyl acetate and repeated collection of the hydrogenous phase. Remaining cell debris was removed by a final centrifugation at 16,000 x *g* for 5 min, the supernatant containing peptides was dried in vacuum centrifuge, and resuspended in 20 µl 0.1% formic acid. Tryptic peptides were analyzed with a Dionex UHPLC (Thermo Scientific) coupled by a nanoelectrospray ion source to an Orbitrap Fusion LC‐MS/MS system (Thermo Scientific). Samples were loaded on a 2 cm C18 trap column (Acclaim PepMap100, Thermo Scientific) and separated using a 150 min non-linear gradient (3–95% acetonitrile/0.1% formic acid, flow rate 300 nl/min) on a 50 cm C18 analytical column (75 µm i.d., PepMap RSLC, Thermo Scientific). Full mass spectrometry scans were acquired with a resolution of 120.000 (full width at half maximum) in the Orbitrap (m/z range 350‐1550). Peptides were fragmented by higher-energy collisional dissociation (HCD, 30% collision energy), and fragment ion spectra were acquired in the order least to highest intensity during a 3‐second maximum cycle time in the ion trap in rapid mode using quadrupole isolation. The following conditions were used: spray voltage of 2.2 kV, heated capillary temperature of 275°C, S‐lens RF level of 60%, a maximum automatic gain control (AGC) value of 1 × 10^6^ counts for MS1 with a maximum ion accumulation time of 50 ms and a maximum AGC value of 3 × 10^3^ for MS2, with a maximum ion accumulation time of 250 ms. A dynamic mass exclusion time window of 30 seconds was set with a 10 ppm maximum mass window.

### Protein identification and quantification

RAW files from mouse samples were searched against the UniProt mouse proteome and the *Aspergillus fumigatus* strain CEA10 proteome (versions March 2018 from UniProt). Patient samples were analyzed using the UniProt databases (homo sapiens version 05.2016, reviewed sequences) and an *Aspergillus fumigatus* Uniprot database (containing reviewed and unreviewed sequences from December 2016) with MaxQuant version 1.5.5.1 (Max Planck Institute of Biochemistry, [22]). The parameters were set as follows: main search peptide tolerance: 4.5ppm; enzyme: trypsin, max. 2 missed cleavages; static modification: cysteine carbamidomethylation; variable modification: methionine oxidation. PSM (peptide-specific matches) and protein FDR were set to 0.01. For advanced identification, the Second Peptide Search in MS2 spectra in both calculations and the Match Between Runs feature for mouse samples were enabled. Hits in either database were manually compared with the other database, yielding the same result. Label-free quantification of proteins with normalization was done in MaxQuant [23]. LFQ minimum ratio count was set to one. Only unique and razor peptides, unmodified or modified, were used for quantification. LFQ protein intensities were then loaded into the Perseus framework (Version 1.6.2.2, Max Planck Institute of Biochemistry [24]). Known contaminants and reverse identified proteins were discarded. Proteins with less than five identifications in at least one group were removed from the data set. Intensities were log_(2)_ – transformed and missing values were imputed from the normal distribution of the data set (width: 0.3, downshift 1.8). Two-sample T-test was used to calculate statistical differences of protein abundances in the different groups. P-values were adjusted according to Benjamini and Hochberg [25] and proteins demonstrating at least a two-fold expression difference and an adjusted p-value <0.05 were considered to be significantly changed in abundance. In addition, for the human BAL samples significant differentially expressed proteins had to be identified with at least two different peptides.

The mass spectrometry proteomics data have been deposited to the ProteomeXchange Consortium via the PRIDE [26] partner repository with the dataset identifier PXD016664.

### Data analysis and visualization

We used the information on differentially abundant proteins (with an adjusted p-value < 0.05 and a fold change ≥ 2) for enrichment analysis and for comparisons between humans and mice. We confirmed that the human samples did not cluster according to sex, steroid treatment, or disease using the tool PCAGO [27]. Gene ontology (GO) and pathway enrichment analysis was based on InnateDB [28]. Only results for GO terms and pathways with a false discovery rate (FDR) ≤0.05 were further considered, excluding all terms with only one and more than 500 genes. Revigo [29] was used to visualize and identify GO terms that are interdependent. This information was used for a clearer GO representation. Therefore, FDR values from GO terms were used as input for Revigo, with an additional adaption of the GO term size database (either *Homo sapiens* or *Mus musculus*). The up- and down-regulated GO terms were manually inserted into the downloaded map of GO terms from Amigo2 [30].

## Results

### Proteomic analysis of human BAL samples

**Figure 1.**
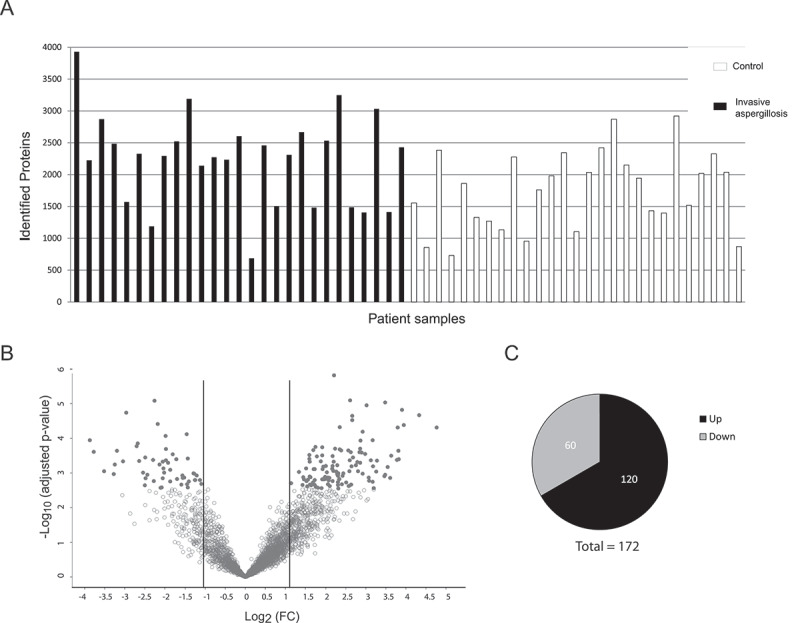
**Detection of proteins in human BAL samples by LC-MS/MS analysis**. (a) Total numbers of identified proteins in each patient sample. (b) Volcano plot of proteins with different abundance in infected versus non-infected samples. The adjusted *p*-value is plotted against the expression fold change of all detected proteins within the different groups. Data points in the lower center area of the plots (empty circles) display unchanged or proteins with no significant fold change. Data points in the upper quadrants indicate proteins (filled circles) with significant negative (left) and positive (right) changes in protein abundances, respectively. (c) Graphical overview of the numbers of proteins with increased abundance (Up) and decreased abundance (Down) in IPA patients compared to the control group.

**Figure 2.**
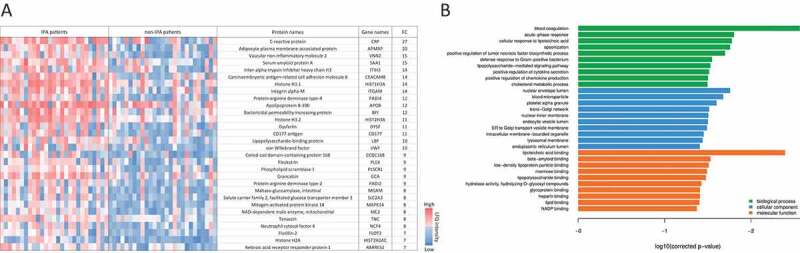
**Proteins with most significant differential abundance determined by LC-MS/MS analysis in human BAL samples**. (a) Heat map of the 30 most abundant proteins during IPA. Levels of proteins with significantly increased abundance in each patient sample are visualized by LFQ intensities with a significant fold change >2 of the IPA group vs non-IPA group. FC values are fold changes. (b) The most significantly overrepresented GO terms of highly abundant proteins for biological process, molecular function and cellular component were determined with an adjusted *p*-value < 0.05. A full list of overrepresented proteins and GO terms are provided in Supplementary Tables 2 and 3.

We identified the lung proteomic signature of IPA by comparing infected patients with controls. BAL samples from 27 patients with probable (n = 23) or proven (n = 4) IPA (according to the 2008 EORTC/MSG criteria [31]) and 27 controls that tested negative for IPA were included in the final analysis. All patients had various underlying diseases and medical treatments (Table 1, Supplementary Table 1). The galactomannan index for BAL samples for all IPA-patients was higher than 1.6 while it was under 0.3 for all non-IPA patients (Table S1). Antibiotic treatment was given to three patients in the non-IPA group and to 21 IPA patients before the bronchoscopy was performed (Table 1, Supplementary Table 1). Five patients of the non-IPA group were found to have bacterial lung infections (caused by *Streptococcus pneumoniae, Enterococcus faecalis, Pseudomonas aeruginosa, or Haemophilus influcencae*) and BAL samples were positive for CMV (Cytomegalovirus) in three samples. Among the IPA group bacteria were identified in three samples and CMV was detected in six samples. A total of 17 patients of the IPA group and 21 patients of the non-IPA group received steroid treatment. Large-scale MS-analysis of the human BALs identified ≥3900 proteins across all samples, with a range of 680 to 3930 detected proteins per sample. The variations were relatively equally distributed between the patient groups (Figure 1(a)). Altogether, the average number of identified proteins was 2240 (± 720) for the IPA and 1760 (± 611) for the non-IPA group. For quantitative analysis, the LFQ (label-free quantification) intensities of proteins in IPA and control samples were analyzed and relative distributions depicted in a volcano plot (Figure 1(b)). In total, 172 proteins were significantly differentially expressed in the IPA group (120 proteins of higher and 52 proteins of lower abundance in IPA BAL fluid (Figure 1(c), Supplementary Table 2). The 30 most highly abundant proteins in IPA are listed in Figure 2 and abundance is visualized in a heat-map for all patients as LFQ intensities. While we found significant over-representation of proteins between the IPA and the control group, we did not observe clustering of distinct candidates in the global proteome that allowed differentiation of IPA cases and controls using Principal Component Analysis (data not shown). Among the most abundant proteins that were enriched in IPA patients were acute phase proteins serum amyloid protein (Saa1) and C-reactive protein (CRP), proteins such as von Willebrand factor (VWF) involved in hemostasis, infection recognition factors such as NLR family CARD domain-containing protein 4 (NLRC4) and Toll-like receptor 2 (TLR2), histones H3.1, H3.2 H3.3 and H2Atype 2-C and proteins of neutrophils such as CD177 antigen, Carcinoembryonic antigen-related cell adhesion molecule (CEACAM) 8, Neutrophil defensin 3 (DEFA3) and Neutrophil cytosol factor 4 (NCF4) (Figure 2(a), Supplementary Table 2). Gene ontology (GO) enrichment analysis of biological process demonstrated enrichment of inflammatory immune responses, including blood coagulation, acute phase response, cellular response to lipoteichoic acid, opsonization processes, and positive regulation of tumor necrosis factor biosynthetic process (Figure 2(b), Supplementary Table 3). Enriched GOs of cellular components included GO annotations mainly involved in transport (ER and Golgi compartments), nuclear envelope lumen, and blood microparticles. Enriched GOs covering molecular function had mainly host factor binding properties (lipoteichoic acid, beta amyloid, mannose, lipopolysaccharide).

**Table 1.** Demographic and clinical characteristics of IPA and non-IPA patients.

	IPA patients (*n* = 27)	non- IPA patients (*n* = 27)
Demographic variables		
Age	58 (±16)	53 (±19)
Sex ratio (male/female)	14/13	13/14
Underlying disease		
Acute Leukemia	2	0
Allo-HSCT	6	0
Influenza	2	0
Lung disease	4	3
Solid tumor	1	1
SOT	11	20
Other disease	1	3

SOT: solid organ transplants, Allo HSCT: allogeneic hematopoietic stem cell transplantation.

Generally, our comparative protein analysis of IPA and non-IPA patient groups revealed differences suggesting that host proteins might be valuable markers to aid diagnosis of aspergillosis. However, despite the advantage of using individual patient samples for the determination of protein levels, we cannot exclude that underlying differences between the two patient cohorts other than aspergillosis, for example the presence or extend of neutropenia, affected the results. While the number of samples in this study allowed to identify common characteristics of the host proteome during IPA, a considerably larger cohort of patients would be necessary to perform analyses that allow identification of specific responses depending on underlying diseases, or the impact of specific therapeutic interventions.

### Proteomic profile of murine BAL fluids

**Figure 3.**
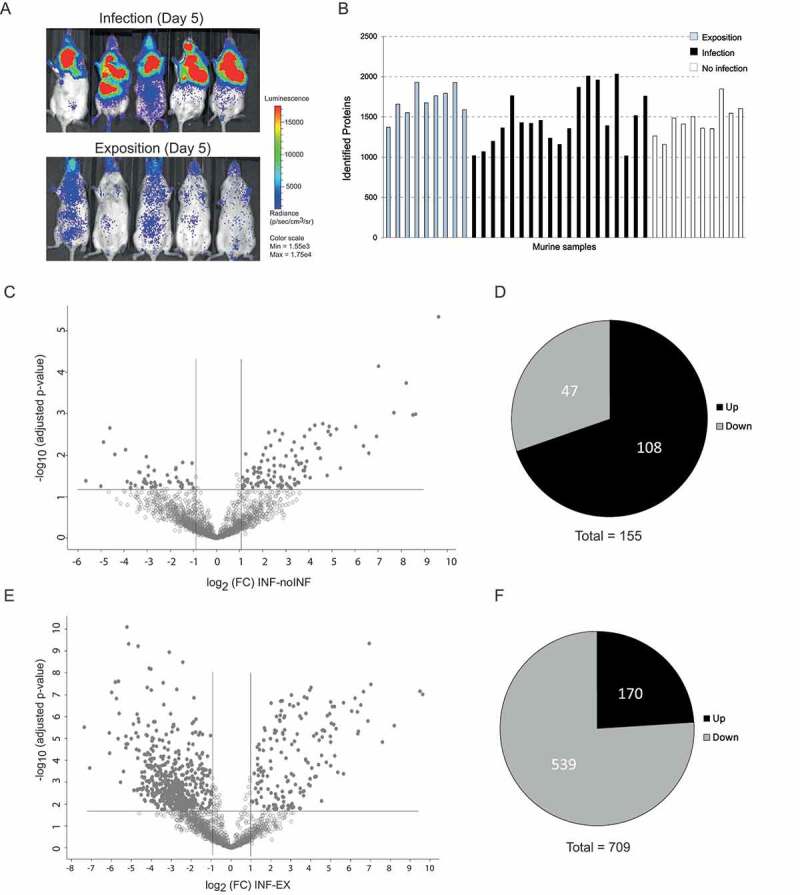
**Detection of proteins in murine BAL samples after pulmonary infection with *A. fumigatus* by LC-MS/MS analysis**. (a) Fungal infection of murine lungs visualized by *in vivo* imaging in five exemplary mice of the infection group versus five mice of the exposition group. (b) Total numbers of identified proteins in each mouse sample. (c) Volcano plot of differentially expressed proteins from infected versus non-infected mice. The adjusted *p*-value is plotted against the abundance fold change of all detected proteins within the different groups. Data points in the lower center area of the plots (gray) display unchanged or proteins with no significant fold change. Data points in the upper quadrants indicate proteins (filled circles) with significant negative (left) and positive (right) changes in protein abundances, respectively. (d) Graphical overview of the numbers of proteins with increased (Up) and decreased (Down) abundance in samples from infected mice compared to non-infected mice. (e) Volcano plot of differentially abundant proteins from infected versus exposed mice. The adjusted *p*-value is plotted against the abundance fold change of all detected proteins within the different groups. Data points in the lower center area of the plots (gray) display unchanged or proteins with no significant fold change. Data points in the upper quadrants indicate proteins (filled circles) with significant negative (left) and positive (right) changes in protein abundances, respectively. (f) Graphical overview of the numbers of proteins with increased (Up) and decreased (Down) abundance in samples from infected mice compared to exposed mice.

**Figure 4.**
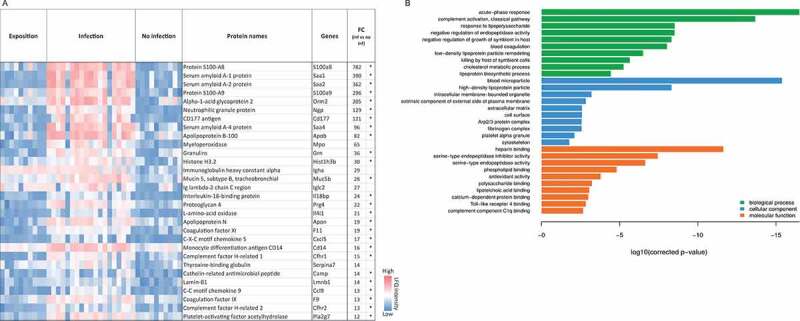
**Proteins with most significant differential abundance in infected versus non-infected mice determined by LC-MS/MS analysis**. (a) Heat map of the Top 30 abundant proteins during IPA. Abundance of significantly upregulated proteins in each animal sample are visualized by LFQ intensities with a significant fold change (FC) >2 between infected and non-infected group. The asterisk indicates proteins that were also significantly increased in comparison to the exposed group in comparison to the exposed group. (b) The most significantly overrepresented GO terms of highly abundant proteins for biological process, molecular function and cellular component were determined with an adjusted *p*-value. A full list of proteins with significantly differential abundance is provided in Supplementary Table 4.

To prevent experimental variation due to confounding human differentials, such as underlying diseases, medications, and possible co-infections, we additionally analyzed BAL samples from a well-established leukopenic murine IPA model. This furthermore allowed comparison of non-exposed (NI), exposed (EX), and infected (INF) groups. The exposure group (EX) was immunocompetent and exposed to *A. fumigatus* spores intranasally, mimicking healthy individuals that inhale *Aspergillus* spores. A second control group (NI) was rendered immunocompromised but was not challenged with *Aspergillus*. The third group (INF) received immune-suppressive treatment and was infected with fungal spores leading to IPA. Within 3–5 d, all animals of the INF group developed invasive aspergillosis, displaying fungal growth by in vivo imaging and clinical symptoms such as dyspnea; however, no fungal growth was detected for the EX group (Figure 3(a)). NI animals remained healthy throughout the study, and no lung alterations were observed upon necropsy. Infected animals were sacrificed for BAL collection when humane end points were reached (d 3–5) while all non-infected and exposed mice were asymptomatic and sacrificed 7 d after inhalation of PBS or spores, respectively. Plating of BAL samples yielded *A. fumigatus* colonies INF BAL, whereas no growth was observed for samples derived from EX or NI mice. In total, 38 out of 40 samples (EX: n = 9; INF: n = 19; NI: n = 10) were successfully measured and included in the analysis. Measurement of two samples yielded extremely low numbers of peptides detected, likely due to technical issues during sample preparation, and were therefore excluded from the analysis. The proteomic profile of the murine pulmonary fluids was examined by LC-MS/MS as performed for human BAL samples. The number of identified proteins in individual samples ranged from 1019 to 2034 per specimen (Figure 3(b)). Variations were equally distributed within the three groups with an average of 1695 (± 181) proteins for EX, 1477 (± 333) for INF, and 1453 (± 192) proteins for the NI group. For quantitative analysis, the LFQ intensities of proteins in INF and NI samples were analyzed, and relative distributions depicted in a volcano plot (Figure 3(c,e)). Quantitative comparison of the INF to the non-infected NI group showed increased abundance of 108 proteins and decreased abundance of 47 proteins (Figure 3(d), Supplementary Table 4), while 170 proteins were increased- and 539 proteins decreased in INF compared to EX (figure 3(f)). This likely reflects the lack of protective immune cells in leukopenic mice (INF and NI). A total of 26 of the 30 proteins most highly abundant in INF compared to NI were also significantly enriched compared to EX (Figure 4(a)). This core set consisted of proteins involved in complement activation, acute phase response proteins such as serum amyloid proteins (Saa1, Saa2, Saa4), alpha-1-acid glycoproteins (Orm1-3) and CRP, antibodies, proteins promoting blood coagulation (coagulation factors 9–11), and protease inhibitors (Figure 4(a,b)). Comparison of proteins enriched in IPA patients and infected mice identified eleven proteins with significantly increase abundance in both hosts, human, and mouse, compared to their uninfected controls (Table 2). These included Fibrinogen Beta chain, serpin Family A member 10, apolipoprotein B, lamin B1, lipopolysaccharide-binding protein, C-reactive protein, inter-alpha trypsin inhibitor Heavy chain 3, and the CD177 molecule (Table 2). Comparison of enriched GO-terms in humans and mice with IPA revealed 8 GO terms enriched in both hosts associated with molecular function and 20 under the term biological processes. These were related to immune recognition (Toll-like receptor cascades and complement cascade) and host response pathways such as blood coagulation (platelet degranulation and aggregation, clotting cascade, cell–surface interactions at the vascular wall), acute phase response, cellular response to lipoteichoic acid and peptidoglycan (Supplementary Table 5).

**Table 2.** Common host proteins that were significantly upregulated during IPA in both human and mice.

Name	Gene	FC in human	FC in mice
Apolipoprotein B	APOB	12	82
CD177 molecule	CD177	11	121
C-Reactive Protein	CRP	27	782
Fibrinogen Beta Chain	FGB	5	3
Hexose-6-Phosphate Dehydrogenase/Glucose 1-Dehydrogenase	H6PD	4	10
Histone H3.1	HIST1H3A	14	12
Inter-Alpha-Trypsin Inhibitor Heavy Chain 3	ITIH3	14	3
Lamin B1	LMNB1	5	14
Lipopolysaccharide Binding Protein	LBP	10	5
Protein Z-dependent protease inhibitor	SERPINA10	3	3
Serum amyloid protein-A1	SAA1	15	390

FC values are fold changes calculated from measured LFQ values.

### Detection of fungal antigens

Our global proteome analysis of BAL samples did not only allow a detailed examination of the host protein profile during IPA but also the identification of fungal proteins that are produced specifically during infection and might serve as valid markers to define fungal infection in the lung. For this, all proteins that were identified with at least two unique peptides were included. Surprisingly, we detected fungal proteins in all animal groups: five proteins were detected in mice that belonged to the exposition or non-infection group and thus seem to be present in murine lungs without infection by *A. fumigatus*. Three of these five proteins, a ribosomal peptide synthase (M11), a Hybrid NRPS/PKS enzyme (M12), and an RNase L inhibitor (M17), were found uniformly in all three groups. The two others, Allergen Asp F4 (M8) and a Beta – alanine synthase (M18), were more frequently found in INF-mice compared to NI and EX. However, the majority of detected proteins (16 out of 21) were exclusively identified in mice with IPA (Table 3). The most abundant infection-specific fungal protein was an uncharacterized protein, M1, with unknown function (Table 3). Interestingly, this protein shared two detected peptide sequences with another fungal protein abundant in BAL samples of infected mice, M4. M1 contains a bacterial SH3 domain in its N-terminal region and shares homologies with M4 and also M2, two proteins that carry an NlpC/P60-like cell-wall peptidase domain, and may therefore have similar activity (Supplementary Figure 1). Despite their abundance in the murine BAL samples, these proteins are normally not produced under laboratory culture conditions [3,8] with exception of M4 that was detected in the *Aspergillus fumigatus* secretome under acidic conditions [32]. Interestingly, the genes for all three proteins were shown to be transcriptionally upregulated during murine IPA [9,33]. We also identified several allergens, AspF2 (M6), Crf1 (M7), and AspF4 (M8), which were predominantly detected in infected mice and have previously been identified in Allergic bronchopulmonary aspergillosis (ABPA) patients as strong immunogenic fungal antigens (Table 3) [34–37]. Furthermore, secreted proteins presumably involved in defense mechanisms (i.e. against oxidative stress and immune stimulation), nutrient acquisition in cell wall maintenance (M20, M21) [8,32,35,38] were identified exclusively in BAL fluids of infected animals (Table 3). Several of these proteins (M10, M13, M21) have previously been shown to be secreted in in vitro culture, and glutamate dehydrogenase (M9) was previously identified as antigenic protein. A signaling protein (M19) and pectate lyase E (M14) have not yet been identified in the fungal secretome but they both carry a signal peptide and were shown to be transcriptionally upregulated in vivo [9,33]. In contrast to murine samples, only few peptides matching fungal proteins were detectable in human BAL, possibly due to higher dilution. In human samples the detection of fungal proteins was also highly unspecific: we identified 12 fungal proteins, including 10 that were detected in both, IPA and control cases (Table 4). Detection of the proteins in uninfected patients and animals could be due to the ubiquitous presence of fungal spores that can be taken up in food products and by inhalation. This illustrates the problem of finding infection-specific fungal biomarkers for invasive pulmonary aspergillosis. These were proteins involved in protein biosynthesis, including several chaperones (the mitochondrial Hsp70, T-complex protein subunit epsilon), a 60S ribosomal protein L30, a highly abundant C6 transcription factor, and MYB DNA-binding domain protein (H4, H5, H9, H11, H12). A positive immune response to mitochondrial Hsp70 was also reported in a study using pooled ABPA patient sera with IPA [7]. Two proteins were detected exclusively in a small subset of the IPA cohort: the allergen AspF2 (H1) and a fungal 14-3-3 family protein (H2) (Table 4).

**Table 3.** Fungal Proteins identified in BAL of mice.

Protein Number	Peptide counts (all)	Razor + unique peptides	Protein ID	Fasta header	Gene	Mice EX INF NI (*n* = 9) (*n* = 19) (*n* = 10)			Signal peptide	Description in other studies
Unknown function										
M1	5	5	B0Y9E0	SH3b domain-containing protein	AFUB_080700	0	12	0	+	32
M2	3	3	B0YAY0	NlpC/P60-like cell-wall peptidase, putative	AFUB_086210	0	9	0	+	32, 33
M3	8	8	B0YEL6	Uncharacterized protein	AFUB_099610	0	5	0	+	
M4	4	2	B0Y269	NlpC/P60-like cell-wall peptidase, putative	AFUB_061470	0	4	0	+	31,32, 33
M5	2	2	B0Y402	Uncharacterized protein	AFUB_056040	0	2	0		
Allergen										
M6	2	2	B0Y6E9	Major allergen Asp F2	AFUB_066690	0	4	0	+	31,32,33,36,37,59,66
M7	4	4	B0XNL0	Extracellular cell wall glucanase Crf1/allergen Asp F9	AFUB_015530	0	3	0	+	31,34,36,37,8, 35
M8	7	7	B0XUQ5	Allergen Asp F4	AFUB_020900	2	10	1	+	36, 37
Defense mechanisms										
M9	2	2	B0Y5K4	Glutamate dehydrogenase	AFUB_063700	0	4	0		66, 7
M10	3	3	B0Y818	Thioredoxin reductase GliT	AFUB_075790	0	3	0		31, 34
M11	5	5	B0Y1Q1	Nonribosomal peptide synthase, putative	AFUB_060400	0	4	3		
M12	5	5	B0YAW2	Hybrid NRPS/PKS enzyme, putative	AFUB_086030	5	4	5		33
Nutrient aquisition										
M13	3	3	B0XT32	Probable pectate lyase A	plyA	0	3	0	+	8, 31
M14	3	3	B0Y9M8	Probable pectate lyase E	plyE	0	5	0	+	32
M15	2	2	B0Y3V5	Extracellular cellulase CelA/allergen Asp F7-like, putative	AFUB_055560	0	5	0	+	31
M16	2	2	B0Y9F1	Glycosyl hydrolase, putative	AFUB_080810	0	3	0	+	
M17	3	3	B0XQF0	RNase L inhibitor of the ABC superfamily, putative	AFUB_009720	4	5	3		
M18	2	2	B0XSS1	Beta-alanine synthase, putative	AFUB_017390	4	12	4		
Signaling										
M19	3	3	B0Y182	FacC-like extracellular signaling protein, putative	AFUB_049010	0	5	0	+	32, 33
Cell wall maintenance										
M20	5	5	B0YCQ5	SUN domain protein (Uth1), putative	AFUB_091030	0	7	0	+	38
M21	4	4	B0XXF8	Probable glucan endo-1,3-beta-glucosidase	eglC	0	4	0	+	8, 31, 35

[32]: Kale et al. 2017 SciRep, [33]: Mac Donagh et al. 2018 PlosPath: Transcriptome studies: upregulation in vivo

[31]: Sriranganadane et al. 2009 JProt, [59]: Fekkar et al. 2012, JProt; [8]: Wartenberg et al. 2011 IJMM, [35]: Champer et al. 2016 JFungi; Proteome studies: detection of secreted proteins

[7]: Virginio et al. 2014 IJMS, [34]: Bacher et al. 2014 JImmun, [36]: Crameri 1998 Int Arch Allergy Immunol, [37]: Singh et al. 2010 JProtRes, [66]: Teutschbein et al. 2016 JProtRes: Immune Proteome Studies: Antigenic protein detected by patient sera

[38]: Gastebois et al. 2013 JBC; study on SUN proteins: Transcriptome study: upregulation during mycelial growth.

**Table 4.** Fungal proteins detected in human BAL fluid by LC-MS/MS.

Protein number	Peptide counts (all)	Razor + unique peptides	Protein IDs	Fasta header	Gene	Patients IP (*n* = 27) non IPA (*n* = 27)	
H1	3	3	P79017	Major allergen Asp f 2	AFUA_4G09580	2	0
H2	5	2	Q4WND4	14-3-3 family protein	AFUA_6G06750	3	0
H3	2	2	Q4WQP9	Gamma-glutamyltranspeptidase	AFUA_4G13580	6	2
H4	5	5	Q4WE46	MYB DNA-binding domain protein	AFUA_5G01730	5	2
H5	2	2	Q4X1H5	Mitochondrial Hsp70 chaperone (Ssc70), putative	AFUA_2G09960	4	2
H6	2	2	Q4WLD0	Polyprenyl transferase pyr6	pyr6	1	3
H7	3	2	Q4WHW6	Serine/threonine-protein phosphatase	AFUA_2G03950	9	4
H8	5	5	Q4X0G8	Fermentation associated protein (Csf1), putative	AFUA_2G1352	2	6
H9	2	2	Q4X1P9	60S ribosomal protein L30, putative	AFUA_2G09200	9	6
H10	4	4	Q4WQU7	Glycosyl transferase, putative	AFUA_4G14070	5	8
H11	3	2	Q4WN57	T-complex protein 1 subunit epsilon	AFUA_6G07540	9	9
H12	2	2	Q4WR07	C6 transcription factor, putative	AFUA_4G14680	11	19

List of all fungal proteins that were identified in individual patient samples with at least two unique peptides.

## Discussion

In this study, we performed a comprehensive analysis of host protein changes during IPA in human and mice and compared protein profiles in both hosts to allow a definition of particular host biomarkers that are specifically enriched in samples positive for IPA.

Despite variations in underlying diseases and medical treatments in humans, we identified host proteins that were overrepresented in IPA versus non-IPA-patients. However, analysis of the more homogenous animal groups yielded a more distinct differentiation of IPA and controls (q-values 2x10^−3^- 4 × 10^−2^ in human vs. 4x10^−8^- 5 × 10^−2^ in mice). It should be noted that we did not perform experimental validation of differential protein abundance by analyzing gene expression due to limited sample volume. However, the majority of proteins found to be differentially abundant in this study were either also identified in other studies, as discussed be low, or in the case of fungal proteins the corresponding genes were shown to be differentially expressed *in vivo* (Table 3 and discussed below).

The significant enrichment of proteins involved in blood coagulation, acute phase response, and cellular response to lipoteichoic acid was not surprising as angioinvasion during IPA leads to pulmonary hemorrhage and has been demonstrated previously in blood and lung samples in rats infected with *Aspergillus* [39]. Increased levels of haptoglobin, an acute phase protein during angioinvasion, were reported in mammals with IPA and since the protein was detected in BAL fluid as well as in serum of leukopenic rabbits, it was proposed as a candidate-circulating biomarker for IPA [16,40,41]. However, although haptoglobin was significantly elevated in leukopenic IPA mice this was not discovered in human BAL samples. In humans, we observed strong expression of the acute phase protein in all patient samples. This likely reflects the induction of haptoglobin in non-IPA patients as a result of the underlying diseases, as the haptoglobin is known to take part in various immune processes and elevated levels have been reported for several types of solid tumors [42,43].

While we identified over 100 host proteins that were significantly elevated in human and mice with IPA relative to uninfected controls, only 11 proteins were overrepresented in both hosts (Table 2). This included several acute phase proteins associated with the degree of tissue damage in mammals which are frequently used in diagnostics. One example is CRP that was also found to be prominently increased during aspergillosis in previous studies [44]. Other examples are Saa1, lipopolysaccharide-binding protein (LBP), and Inter-Alpha-Trypsin Inhibitor Heavy Chain 3 (ITIH3). Similar to CRP, Saa and LBP are known to be induced during bacterial infections and infection with *Candida* species and thus these proteins seem unsuitable for differentiating between fungal and bacterial infection [45,46].

Significantly increased in BAL samples from both hosts were proteins with crucial roles during blood coagulation: Protein Z-dependent protease inhibitor (SERPINA10), an inhibitor of factors Xa and XIa, and fibrinogen beta chain (FGB), an important component of blood clots during vascular injury. Previous studies have shown the expression of fibrinogen-binding proteins on the surface of conidia of *A. fumigatus* which promote binding to fibrinogen and host tissue [40,47]. The release of fungal proteases during infection has been reported to result in fibrinogen cleaving and activation of the TLR4 signaling pathway through binding of fibrinogen cleavage products [48–50]. The airway protease activity promoted by *A. fumigatus* initiates both allergic airway disease and antifungal immunity [48,49].

Apolipoprotein A-1 was previously described as one of the most abundant proteins in BAL samples in a neutropenic rabbit model of IPA [44]. High density lipoproteins containing apolipoprotein A-I are antibacterial [51,52], and apolipoprotein B (ApoB) inhibits the hemolytic activity of *A. fumigatus* Asp-hemolysin [53]. Interestingly, in our study protein levels for ApoB were significantly decreased in both hosts during aspergillosis; additionally, elevated levels for apolipoprotein E and N were detected in mice. Besides inflammatory proteins we observed a strong IPA-associated increase of CD177, a glycosyl-phosphatidylinositol-anchored receptor expressed on circulating neutrophils that contributes to neutrophil transmigration, is upregulated upon neutrophil stimulation, and is elevated during severe bacterial infections in patients [54]. Neutrophils play a prominent role in the host defense against *A. fumigatus*, and neutropenia or defective neutrophil functions are the most prevalent risk factors for IPA [55]. However, the levels of neutrophils were very variable within the human IPA cohort depending on the underlying disease (Supplementary Table 1), and this affected CD177 levels: In patients with severe neutropenia (undetectable neutrophils in four patients) CD177 levels were low, although patients were classified in the IPA group. Patients with very high neutrophil numbers (>10,000/µl) displayed above average CD177 levels despite the absence of IPA. However, within a range of neutrophil concentrations (200–10,000/µl) the CD177 levels were increased in the IPA group (in 14 of 19 IPA-cases).

The comparison of leukopenic mice (infected and non-infected) showed a similar trend of increased CD177 abundance as seen with human samples (in 15 of 19 INF mice). Here, it should be noted that even though animals were leukopenic, low numbers of neutrophils were still present, and these cells were likely recruited to the site of infection. We therefore assume that the overrepresentation of CD177 in both infected humans and mice is a consequence of fungal infection and not the effect of myeloid disorders or treatments, but that CD177 would only be useful as a marker for infection if neutrophil counts are within a certain range.

While we identified proteins that were present in elevated levels in both human and murine IPA samples, a large number of proteins showed significant differences in abundance in IPA and non-IPA samples in one species only. This could be due to clinical conditions and underlying diseases present in human patients (for example hematological malignancy or graft versus host disease), that are not present in the animal model. Furthermore, general differences in murine and human immune responses exist [56]. One example is the recently discovered C-type lectin receptor MelLec, which recognizes fungal melanin and is expressed broadly by cells of the human immune system, whereas it is confined to a specific type of endothelial cells in the mouse [57]. While these differences affect the immune response to *A. fumigatus* on the molecular level, and thus might explain host-specific proteome signatures during IPA, it should however been noted that the pathogenesis and overall immune response in mice and humans with IPA are similar [58].

In previous studies, the search for fungal biomarkers for improved diagnosis of IPA has mainly focused on fungal cultures grown in different artificial growth media such as *Aspergillus* minimal media, YPD, Potatoe dextrose media, and Czapek Dox media [6,8,35]. The fungus is known for its high stress tolerance and for its ability to adapt to various growth conditions. Under energy and metal deprivation, the fungus releases various hydrolases and proteinases in its surrounding environment to degrade polymers for nutrient uptake and it responds to stress such as iron depletion and hydrogen peroxide by changing its transcriptomic profile. The examination of biological samples from infected individuals for their protein content allowed us to identify fungal proteins that are produced during IPA. Among the *A. fumigatus* proteins identified in BAL samples of mice, there were several that are known to be produced and secreted *in vitro* cultures and may generally be important for fungal growth and survival. Examples are the highly immunogenic glucanase Crf1 [34,35], pectate lyase A, glucan endo 1,3- beta glucosidase [8,35], and SUN domain protein [38], that were detected specifically in infected animals in our study. Other proteins specific for BAL from infected animals have only putative or unknown functions. Interestingly, three of the most abundant proteins with unknown function share sequence homology (Supplementary Figure 1), and the coding genes were found to be transcriptionally upregulated during IPA in mice [9,33]. This could indicate that these proteins are beneficial for *Aspergillus* during infection, but this remains to be tested.

While several fungal allergens were detected in BAL fluids, AspF2 was the only allergen detected in both murine and human samples and was exclusively found in the infected groups (Tables 3 and 4). AspF2 has previously been identified by Difference Gel Electrophoresis in co-localization with ApoE in enriched sera of leukopenic mice [59]. There are several reports of the detection of AspF2 in ABPA and AspF2 is recognized by specific IgE antibodies in these patients [60,61]. AspF2 was recently shown to recruit several plasma regulators, Factor H, Factor H-like protein, and plasminogen, thereby blocking host immune attack [62]. AspF2 was previously shown to be induced at low zinc levels [63]. One of the factors that may add to a decrease of zinc in infected lungs might be the presence of calprotectin. This protein complex, formed by S100A8 and S100A9, was highly enriched in infected murine lungs and is known to affect *A. fumigatus* growth by chelating zinc and manganese [64]. However, despite its possibly important biological role for antifungal defense calprotectin does not seem to be a suitable marker for IPA since the protein complex was detected in high amounts in all human samples of both infection and control groups. The detection of AspF2 in both, humans and mice with IPA, suggests that it could be used as biomarker for IPA diagnosis in addition to established markers such as galactomannan.

Our untargeted proteomics approach identified 16 proteins that were exclusively detected in infected animals. However, none of the proteins were found in all animals of this group, which could be due to the high dilution effect in the samples without enrichment methods. Although the Orbitrap mass analyzer provides high sensitivity there are detection limits, and the high abundance of host proteins might have hampered the detection of low abundance fungal proteins.

We did however also detect fungal proteins in non-IPA patients or non-infected animals. The unspecific detection of fungal antigens is not an uncommon problem in the search for new fungal biomarkers of IPA. For example antifungal therapy has been reported to lead to occurrence of false-positive results in patient specimens using commercial IPA diagnostic tools and detection of anti-fungal antibodies independent of the infection status of patients [65,66]. As in this study *A. fumigatus* could only be cultured from BAL of infected mice, but not exposed or uninfected animals, we can exclude significant colonization or infection of these groups. The reactivity of sera from human control patients to fungal antigens [7,65,67] could indicate either environmental exposure or low-level colonization of the respiratory tract [68] sufficient to raise an immune response despite the lack of infection symptoms. Environmental exposure to fungal proteins present in bedding or chow, or colonization below the culture detection limit might also explain the detection of fungal proteins in non-infected mice. Furthermore, false-positive identification of fungal proteins based on limited peptide sequences cannot be excluded.

However, the majority of the fungal proteins selectively detect in Bal samples of infected mice were also identified as upregulated on the transcriptional level and/or detected by patient sera in other studies (Table 3), which validates their *in vivo* expression. Therefore, the newly identified uncharacterized proteins (M1-5) that were detected exclusively in infected mice, and especially the three proteins (M1, M2, and M4) that were shown to be highly upregulated *in vivo* in previous studies [9,33], might be promising candidates as fungal IPA biomarkers.

Our study provides a broad overview of the proteomic profile during IPA. While none of the identified host factors are specific for IPA but might also be induced by other types of infection, a combination of the 11 common factors found in human and murine hosts might be a valuable tool for future diagnostic approaches. The comparison of particular proteins with increased abundance during the pulmonary infection in either host also provides a more detailed understanding of the extent to which data from experiments with mice can be translated to human patients.

The work presented here is to our knowledge the first comprehensive study of the proteomic profile from lung specimens in a larger number of IPA patients without using biological pools for analysis. The detailed examination of BALs from the murine model also allowed us to identify specific fungal proteins that have not been identified under culture conditions. We propose that these proteins are specifically induced during infection and might therefore also be potential candidates as fungal biomarker.

## Supplementary Material

Supplemental Material
