# Supplementary material for: Proteome analysis of bronchoalveolar lavage fluids reveals host and fungal proteins highly expressed during invasive pulmonary aspergillosis in mice and humans
Source: Virulence. 2020 Oct 12;11(1):1337–51. doi: 10.1080/21505594.2020.1824960 (PMC7549978; doi:10.1080/21505594.2020.1824960)
Supplement: Supplemental Material [file KVIR_A_1824960_SM5847.zip › MachataSupplementary Figure legends.docx]

**Supplementary figures and Tables**

**Supplementary Figure S1:** Protein sequence alignment of proteins M1, M2 and M4 of *A. fumigatus* that were highly abundant in murine BAL samples. The alignment was performed using ClustalW2 **A)** Red columns showing common regions and grey column no homologue areas **B)** In more detail on amino acid level with red characters representing identical amino acids, blue characters similar amino acids and grey no similarities.

**Supplementary Table S1:** Detailed clinical characteristics of IPA and non-IPA patients used for BAL analysis.

**Supplementary Table S2:** Full list of proteins that were differentially expressed in human BAL samples from IPA patients compared to non-IPA patients determined by LC-MS/MS analysis. Expression levels of significantly up/downregulated proteins in each animal sample are visualized by LFQ intensities with a significant fold change >2 of the IPA group vs non-infected group shown as log2 FC value. Proteins were considered that had more than 2 peptides and were detected in at least 5 samples in at least one group (adjusted p values under 0.05).

**Supplementary Table S3:** Enrichment analysis showing a full list of GO-terms of differentially expressed proteins in human BAL samples performed with InnateDB with an FDR < 0.05.

**Supplementary Table S4:** Full list of proteins that were differentially expressed in murine BAL samples from infected mice compared to non-infected mice determined by LC-MS/MS analysis. Expression levels of significantly up/downregulated proteins in each animal sample are visualized by LFQ intensities with a significant fold change >2 of the IPA group vs non-infected group shown as log2 FC value. Proteins were considered that had more than 2 peptides and were detected in at least 5 samples in at least one group (adjusted p values under 0.05).

**Supplementary Table S5:** Common enriched GO-terms and pathways for upregulated proteins during IPA versus non-infected controls in humans and mice.

**Supplementary Table S6: Fungal proteins detected in human BAL fluid by LC-MS/MS.** List of all fungal proteins that were identified in individual mice with peptide counts more than 1 and at least one unique peptide.
