# Supplementary figures and images for: Proteome analysis of bronchoalveolar lavage fluids reveals host and fungal proteins highly expressed during invasive pulmonary aspergillosis in mice and humans
Source: Virulence. 2020 Oct 12;11(1):1337–51. doi: 10.1080/21505594.2020.1824960 (PMC7549978; doi:10.1080/21505594.2020.1824960)

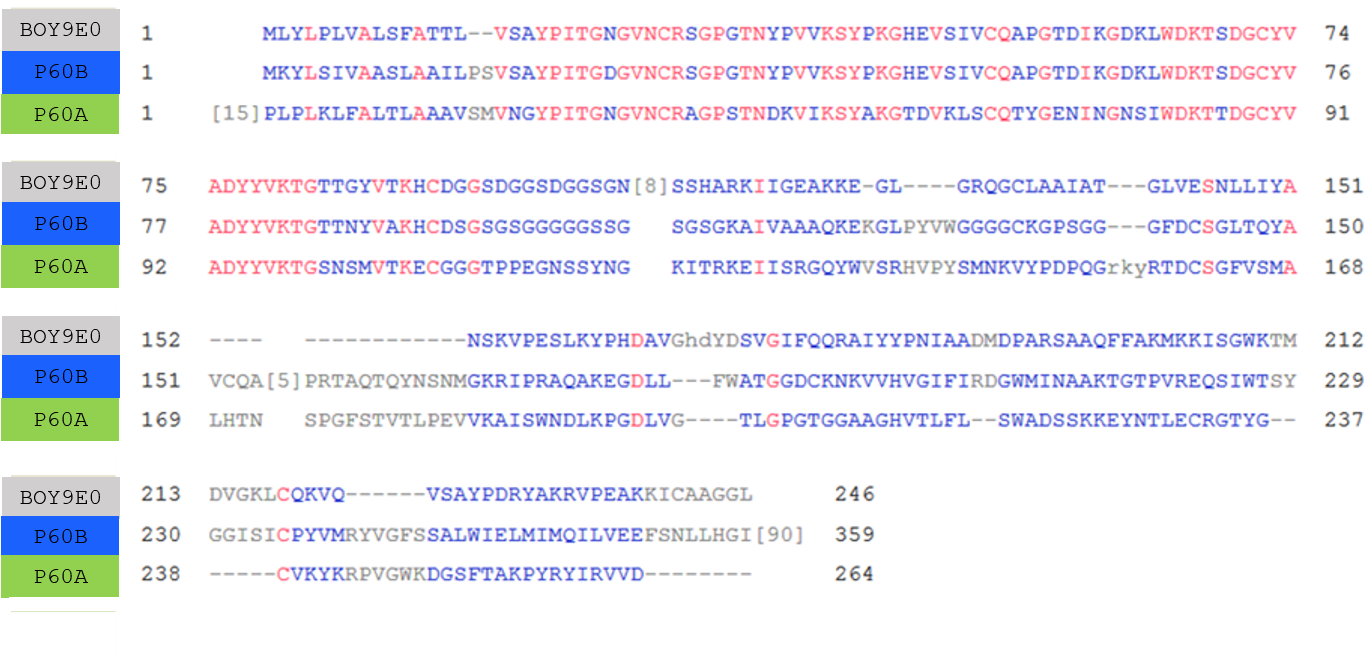


M1

M4

M2

M1

M4

M2

M1

M4

M2

M1

M4

M2


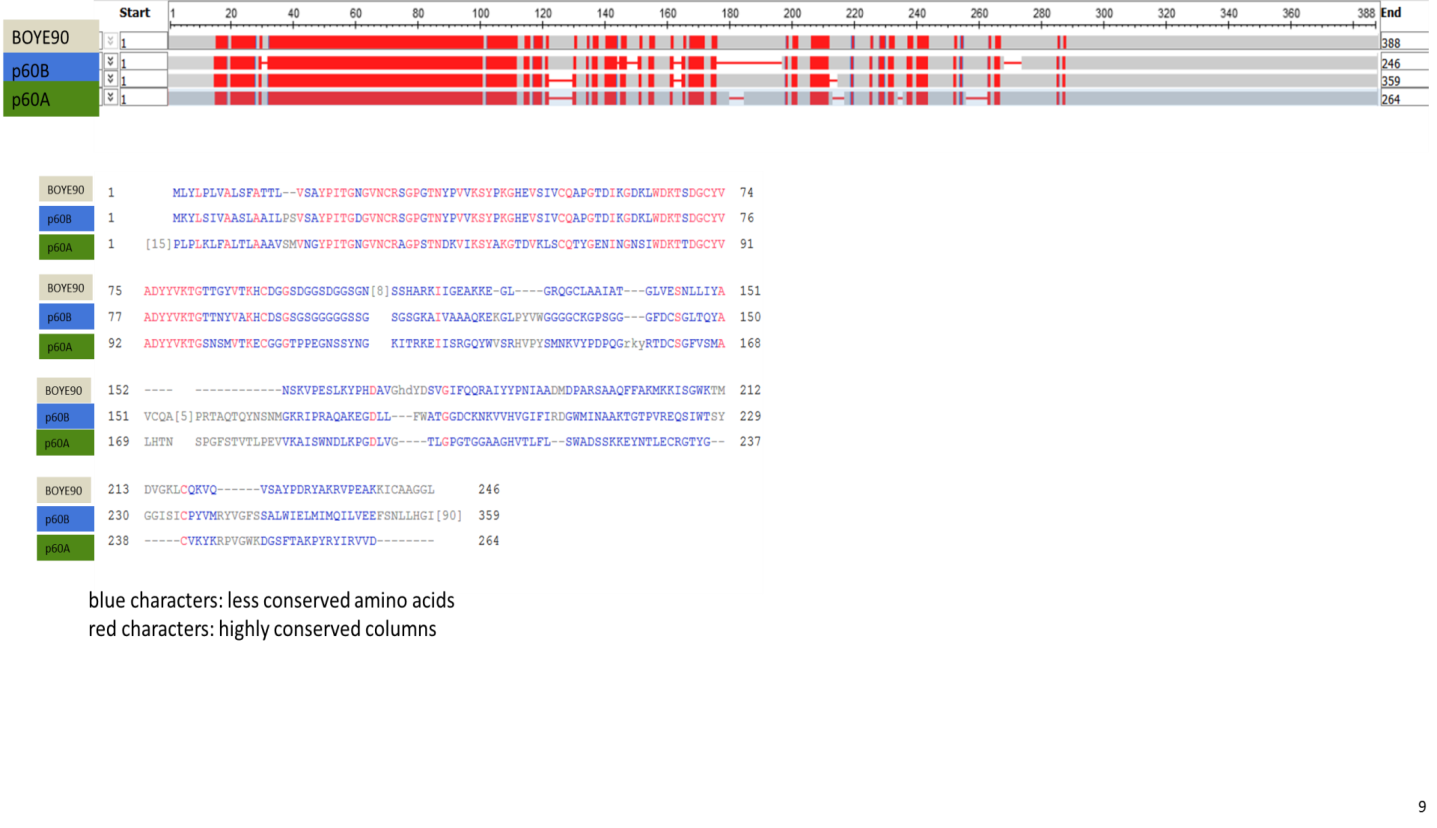


M1

M4

M2

A

B

Supplement: Supplemental Material [file KVIR_A_1824960_SM5847.zip › MachataSupplementaryFigure1.docx]
